# Roles of osteocalcin in the central nervous system

**DOI:** 10.1111/cns.70016

**Published:** 2024-09-09

**Authors:** Xiao‐Shan Qi, Xin He, Ying Peng, Xing‐Hong He, Qian‐Yu Yang, Kai Jiao, Heng Liu

**Affiliations:** ^1^ Department of Radiology Affiliated Hospital of Zunyi Medical University, Engineering Research Center of Intelligent Medical Imaging in Guizhou Higher Education lnstitutions, Medical Imaging Center of Guizhou Province Zunyi China; ^2^ The First Clinical Medical College Zunyi Medical University Zunyi China; ^3^ State Key Laboratory of Military Stomatology & National Clinical Research Center for Oral Diseases & Shaanxi Key Laboratory of Oral Diseases, School of Stomatology The Fourth Military Medical University Xi‘an China

**Keywords:** bone–brain crosstalk, central nervous system, comorbidity, osteocalcin

## Abstract

**Background:**

Bone‐derived protein osteocalcin, which has beneficial effects on brain function, may be a future research direction for neurological disorders. A growing body of evidence suggests a link between osteocalcin and neurological disorders, but the exact relationship is contradictory and unclear.

**Scope of Review:**

The aim of this review is to summarize the current research on the interaction between osteocalcin and the central nervous system and to propose some speculative future research directions.

**Major Conclusions:**

In the normal central nervous system, osteocalcin is involved in neuronal structure, neuroprotection, and the regulation of cognition and anxiety. Studies on osteocalcin‐related abnormalities in the central nervous system are divided into animal model studies and human studies, depending on the subject. In humans, the link between osteocalcin and brain function is inconsistent. These conflicting data may be due to methodological inconsistencies. By reviewing the related literature on osteocalcin, some comorbidities of the bone and nervous system and future research directions related to osteocalcin are proposed.

## Graphical abstract

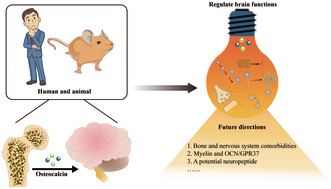

## 1 INTRODUCTION

Osteocalcin, the most abundant noncollagen protein in bone, undeniably plays a role in bone formation and bone loss.^1^, ^2^, ^3^, ^4^ A decade ago, uncarboxylated osteocalcin was shown for the first time to cross the blood–brain barrier and bind specifically to neurons to regulate the central nervous system.^5^ Thus, a pivotal mouse experiment opened a new era of research on osteocalcin‐mediated regulation of the central nervous system.^5^, ^6^, ^7^

Since then, research into the regulation of osteocalcin has focused on the central nervous system. Since most of the studies are based on animal models and there are differences between human and animal osteocalcin, the evidence from human studies is contradictory and inconsistent.^8^ Therefore, it is timely to review the available evidence in light of future studies on the role of osteocalcin in the bone–brain axis. This article reviews the expression and role of osteocalcin in the normal central nervous system and examines the role of osteocalcin in current animal models and human clinical studies. Finally, we discuss the potential limitations of the current research and propose some directions for future research.

## 2 SYNTHESIS AND RELEASE OF OSTEOCALCIN

**FIGURE 1:**
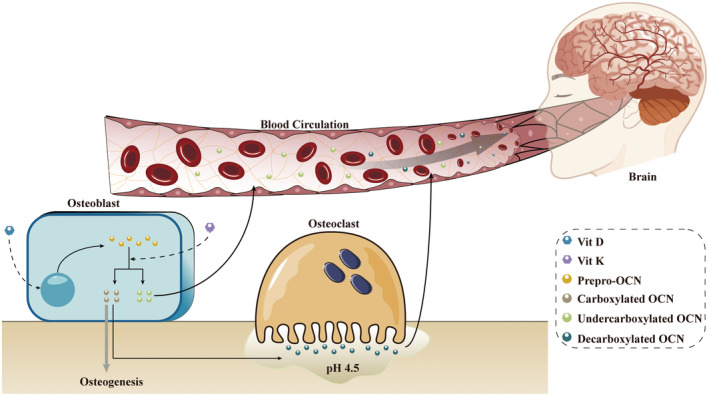
Osteocalcin synthesis and release. The process by which osteocalcin peptides are carboxylated by vitamin K. Two types of osteocalcin can be generated depending on the amount of vitamin K: Carboxylated osteocalcin, which is involved in osteogenesis, and undercarboxylated osteocalcin, which can enter the blood circulation. The decarboxylation of osteocalcin leads to the production of decarboxylated osteocalcin, which can also enter the circulation and participate in acidic bone resorption by osteoclasts. For simplicity, forms of osteocalcin that enter the brain through the blood–brain barrier or placental barrier and thus function in the blood circulation are collectively known as undercarboxylated osteocalcin (with functions similar to uncarboxylated or decarboxylated osteocalcin). Of course, partially intact carboxylated osteocalcin can also be present in the blood circulation, but this is not shown in the figure.

This chapter begins with a brief description of how osteocalcin is synthesized and released into the circulation. In humans, the gene that encodes osteocalcin, *BGLAP*, is located at chromosome 1q25, and its transcription is regulated by vitamin D and *Runx2/Cbfa1* (Figure 1).^2^, ^9^, ^10^, ^11^, ^12^, ^13^ The transcribed peptide chains of *BGLAP* undergo proteolysis followed by vitamin K‐dependent gamma carboxylation at three amino acid residues (glu‐17, ‐21, and ‐24), giving osteocalcin a high affinity for hydroxyapatite and the extracellular matrix.^14^, ^15^ Since these processes occur more frequently during osteoblast maturation, osteocalcin is also a marker of osteoblast differentiation and maturation.^16^, ^17^ Through acidification by the extracellular matrix during osteoclastic bone resorption, osteocalcin deposited in the mineralized bone matrix can be decarboxylated and released into extraosseous organs.^18^ Moreover, bone resorption affects the decarboxylation of osteocalcin.^19^

Modeling different forms of osteocalcin via molecular dynamics simulations revealed that the three α‐helices present in the structure form the hydrophobic core of osteocalcin, which has a compact spherical structure as a whole. One of the special structural features of human osteocalcin is the asymmetric distribution of the positive and negative amino acids in the structure, which produces a surface with positive and negative charges on both sides of the protein. Notably, the differences in the structure and kinetics of different forms of osteocalcin may lead to differences in its biological activity. Therefore, future functional studies will quantify the proposed cellular receptor binding affinity and activation with different forms of osteocalcin, which will help elucidate its specific mechanism of action.^14^

To facilitate further understanding, current experiments have shown that there are two main functional osteocalcin molecules in the body: carboxylated osteocalcin (mainly involved in osteogenesis) and uncarboxylated or undercarboxylated or decarboxylated osteocalcin (mainly in the blood circulation).

## 3 ROLE OF OSTEOCALCIN IN THE NORMAL CENTRAL NERVOUS SYSTEM

### 3.1 Neuronal structure and neuroprotection

**FIGURE 2:**
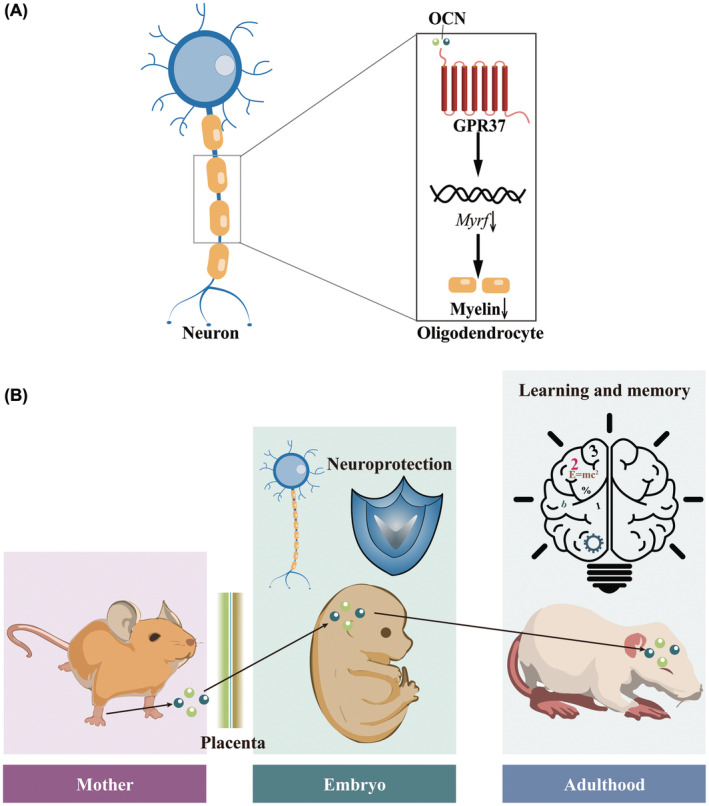
(A) Osteocalcin regulates myelin homeostasis via GPR37. Osteocalcin regulates myelination in oligodendrocytes and affects the thickness of myelin surrounding neurons. OCN, osteocalcin. (B) Effects of maternal osteocalcin on embryos and adult offspring. Maternal osteocalcin can cross the placental barrier, affect neurogenesis in the embryo, and play a neuroprotective role. Maternal osteocalcin affects learning and memory in adult offspring.

Using cell culture models and image analysis, osteocalcin has been shown to promote neurite outgrowth and regulate myelin homeostasis. First, in PC12 cells, both uncarboxylated osteocalcin and carboxylated osteocalcin stimulate NGF‐induced neurite outgrowth; however, this effect is abolished in the absence of osteocalcin.^20^ Second, in oligodendrocytes, osteocalcin (OCN) regulates oligodendrocyte differentiation and myelination via G protein‐coupled receptor (GPR) 37 and maintains the balance between myelin production and degradation in the central nervous system.^7^ There are two important points related to the OCN/GPR37 axis (Figure 2A). First, *Myrf* may mediate signaling via the OCN/GPR37 axis. In both in vivo and in vitro studies, exogenous supplementation with osteocalcin reduced the expression of *Myrf* in a dose‐dependent manner. As GPR37 is not expressed in myelinating Schwann cells, it is an osteocalcin‐specific receptor expressed in mouse oligodendrocytes.^7^

According to studies of different critical periods of biological development, osteocalcin may be linked to the central nervous system. In mice, maternal osteocalcin has been shown to cross the placental and blood–brain barriers, promote neurogenesis, and play a neuroprotective role in the embryonic brain (Figure 2B). The specific evidence that osteocalcin crosses the placental barrier and blood–brain barrier has been excellently reviewed and published.^5^, ^21^ Generally, maternal osteocalcin accounts for the majority of osteocalcin in the embryo; conversely, a lack of osteocalcin in the embryos of mothers with different maternal phenotypes also leads to varying degrees of neuronal apoptosis.^5^ Specifically, on embryonic day 18.5, the embryos of osteocalcin‐deficient (*OCN* ^*−/−*^) mice have twice as many apoptotic cells in the hippocampal region as those of wild‐type mice.^5^

### 3.2 Cognitive function and anxiety

**FIGURE 3:**
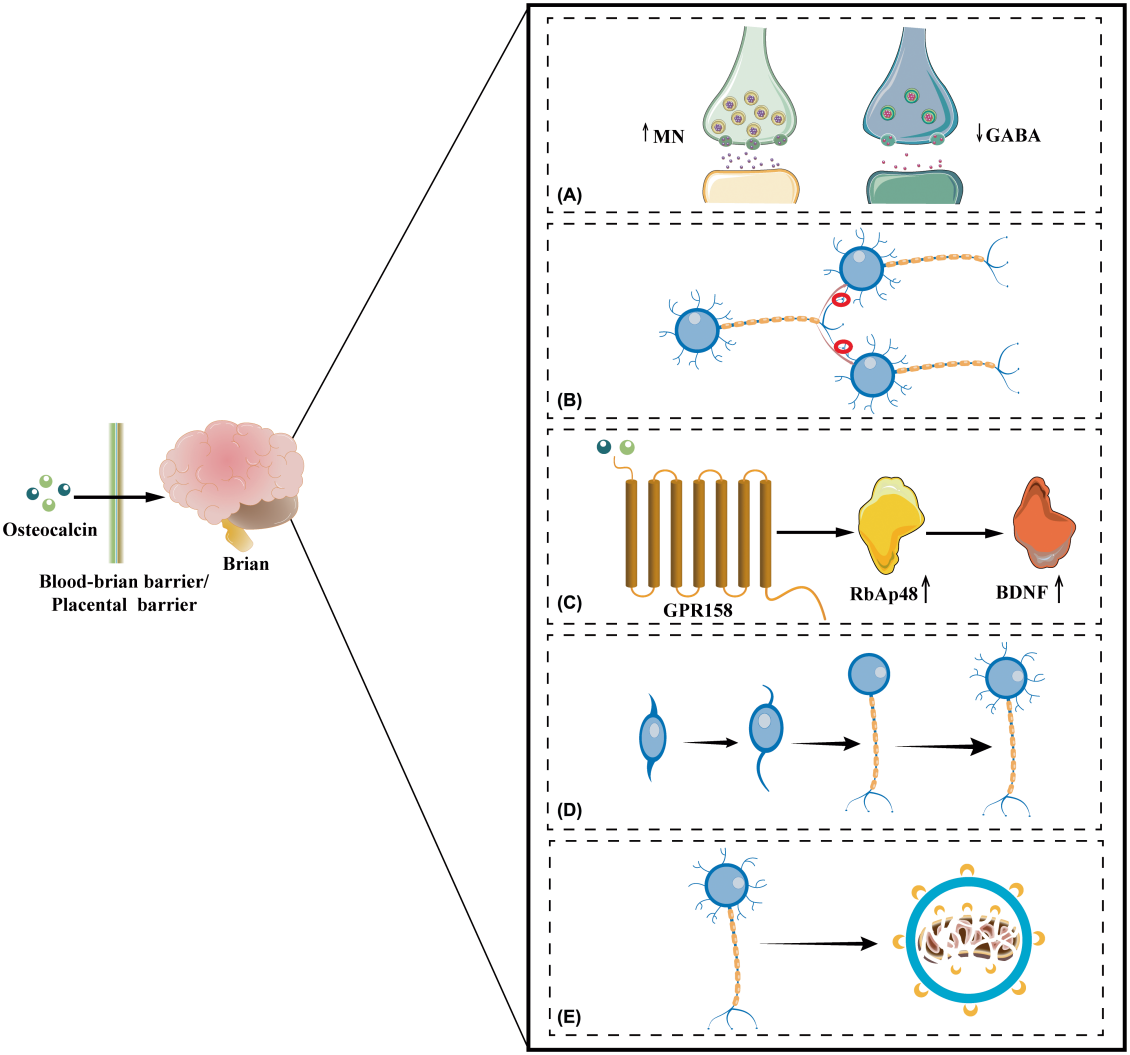
There are five mechanisms by which osteocalcin regulates cognition and anxiety. After crossing the blood–brain barrier or placental barrier, osteocalcin is known to bind to specific neurons in the brain through five mechanisms. (A) Osteocalcin regulates neurotransmitter release, increasing monoamine neurotransmitter release and reducing GABA release. (B) Osteocalcin increases synaptic plasticity. (C) OCN/GPR158 promotes the synthesis of RbAp48, leading to increased BDNF release. (D) Osteocalcin promotes neurogenesis. (E) Osteocalcin promotes neuronal autophagy. BDNF, brain‐derived neurotrophic factor; GABA, gamma‐aminobutyric acid; MNs, monoamine neurotransmitters.

The details of how osteocalcin regulates cognition and anxiety are well summarized, and to avoid repeating the previous review, we briefly summarize the mechanism by which osteocalcin regulates cognition and anxiety.^21^ Osteocalcin regulates a variety of neuronal activities related to cognition and anxiety, such as neurotransmitter synthesis, synaptic plasticity, brain‐derived neurotrophic factor synthesis, neurogenesis, and autophagy (Figure 3).

In mice, *OCN* ^*−/−*^ animals exhibit molecular changes in neurotransmitter synthesis compared with littermate wild‐type mice, leading to decreased release of monoamine neurotransmitters (dopamine, norepinephrine, and serotonin) and increased gamma‐aminobutyric acid release via regulation of the gene expression of the enzymes required for synthesis of these neurotransmitters.^5^ Genetically, maternal osteocalcin is necessary for spatial learning and hippocampus‐dependent memory in adult offspring because it influences adult hippocampal neurogenesis (Figures 2B and 3).^5^ Osteocalcin increases synaptic plasticity and ameliorates age‐related memory loss by regulating GPR158.^6^ OCN/GPR158 interacts with the histone‐binding protein RbAp48, which regulates inositol 1,4,5‐trisphosphate and brain‐derived neurotrophic factor expression.^6^, ^22^ Osteocalcin acts as a direct hormonal inducer of autophagy in hippocampal neurons,^23^ ultimately improving hippocampus‐dependent memory and increasing long‐term potentiation. The relationships between osteocalcin and these targets are shown in Figure 3.

## 4 ROLE OF OSTEOCALCIN IN THE ABNORMAL CENTRAL NERVOUS SYSTEM

In the previous section, osteocalcin was shown to act primarily as a hormone, and its known regulatory functions were reviewed.^24^ However, in the body, osteocalcin is more dynamic, especially in disease states, so we will review the role of osteocalcin in neurological disorders.

### 4.1 The role of osteocalcin in brain function in animal models

At present, there are three animal models of osteocalcin‐related brain dysfunction: cognitive dysfunction, Alzheimer's disease, and Parkinson's disease.

Gu et al. established a rat model of type 2 diabetes mellitus in 2017 with a high‐fat and high‐sugar diet and low‐dose intraperitoneal injection of streptozotocin.^25^ A comparison of the cognitive ability of individuals in the low‐level undercarboxylated osteocalcin group and high‐level undercarboxylated osteocalcin group revealed that a decrease in the serum undercarboxylated osteocalcin level was correlated with cognitive impairment. Sedky et al. showed a dose‐dependent improvement in cognitive function in type 2 diabetes mellitus rats after the administration of anti‐diabetic medication.^26^ The serum osteocalcin level was significantly increased and correlated with improvements in cognitive dysfunction. Zhao et al. also reported that undercarboxylated osteocalcin improved cognitive dysfunction in a dose‐dependent manner.^27^ The rat models of these three studies were all type 2 diabetes mellitus models and had relatively good positive results, but there are still some factors to note: the comprehensiveness of the cognitive assessment methods and the different types of osteocalcin (carboxylated osteocalcin, undercarboxylated osteocalcin, and total osteocalcin).^14^

**FIGURE 4:**
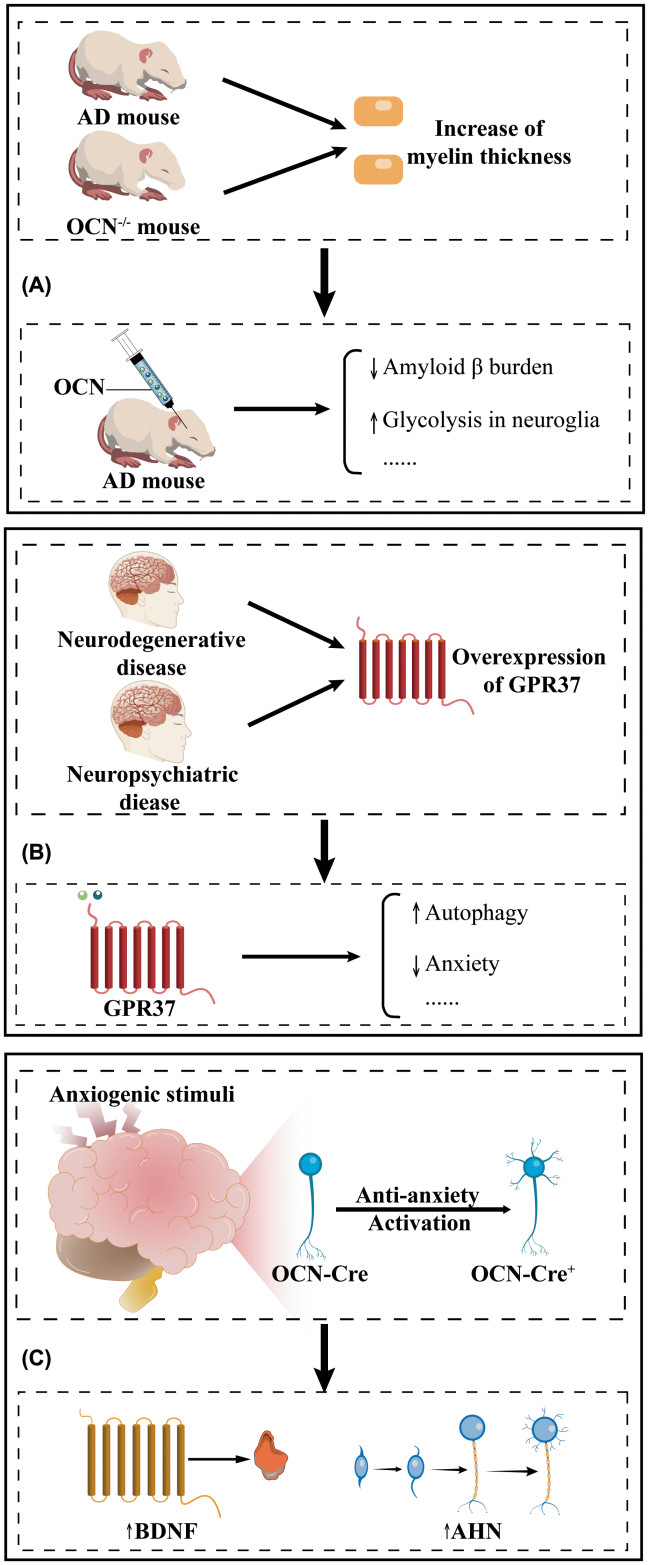
Schematic showing future directions for research on the role of osteocalcin in the central nervous system. (A) Potential mechanisms of osteocalcin in mice with Alzheimer's disease. (B) Potential mechanisms of OCN/GPR37 in neurodegeneration and neuropsychiatric disorders. (C) Osteocalcin is a potential neuropeptide. During anxiety, OCN‐Cre neurons are activated and play an anti‐anxiety role by promoting the expression of brain‐derived neurotrophic factor and adult hippocampal neurogenesis. AD, Alzheimer's disease; AHN, adult hippocampus neurogenesis; BDNF, brain‐derived neurotrophic factor; OCN, osteocalcin.

In an Alzheimer's disease mouse model, Shan et al. proposed that osteocalcin ameliorates cognitive impairment in Alzheimer's disease mouse models by reducing the amyloid β burden and upregulating glycolysis in neuroglia.^28^ Notably, osteocalcin does not improve glycolysis in neurons but rather in astrocytes and microglia. Furthermore, we investigated whether osteocalcin exerts beneficial effects on Alzheimer's disease by binding to GPR158, GPR37, or other receptors (Figure 4).

Guo et al. improved the behavior disorder of a Parkinson's disease rat model induced by 6‐hydroxydopamine after intraperitoneal injection of exogenous osteocalcin.^29^ Osteocalcin was also found to affect glial cells but inhibited the proliferation of astrocytes and microglia. Moreover, this study did not use a GPR158 knockout Parkinson's disease model to determine whether osteocalcin truly exerts its neuroprotective effect through this central receptor in Parkinson's disease rats. According to further research by Hou et al., gut microbiota‐derived propionate mediates the neuroprotective effect of osteocalcin in Parkinson's disease mouse models.^30^ However, this study focused more on the protective effect of osteocalcin on Parkinson's disease mice than on its therapeutic effect. Future research may further explore the therapeutic effect of osteocalcin after Parkinson's disease model establishment.

### 4.2 The association between osteocalcin and brain function in humans

The relationship between blood or cerebrospinal fluid concentrations of osteocalcin and various measures of brain function has been studied in multiple studies in different populations, as summarized in Table 1.

**TABLE 1:** Association of osteocalcin with brain function in humans.

References	Participant characteristics (men/women)	Measurement of brain function and osteocalcin	Findings	Association of osteocalcin with brain function
^32^	n: 225 (108/117) Age: 74 Health Status: Community dwelling	Brain function: CANTAB OCN: CMIA	Plasma OCN levels were positively associated with measures of executive functioning and global cognition scores in the older women	Positively
^36^	n: 44 (21/23) Age: 50 Health Status: Obese and controls	Brain function: IGT tOCN: ELISA	Lower tOCN associated with higher IGT scores	Positively
^34^	n: 196 men Age: 56 Health Status: T2DM	Brain function: RBANS uOCN: ELISA	Serum uOCN was positively correlated with RBANS scores	Positively
^38^	n: 103 (15/88) Age: 80 Health Status: Community dwelling	Brain function: 19 cognitive function tests uOCN & cOCN: ELISA tOCN: ECLIA	No significant association between tOCN and uOCN and the rate of cognition change	No correlation
^33^	n: 42 PM women Age: 58 Health Status: Community dwelling	Brain function: CANTAB tOCN & uOCN: RIA	Serum uOCN predicted tasks associated with reaction time and executive function	No correlation
^41^	n: 95 (47/48) Age: 62 Health Status: T2DM	Brain function: PSS cOCN: ELISA	Higher cOCN associated with higher PSS scores	Negatively
^35^	n: 82 men Age: 70 Health Status: Early‐stage AD and controls	Brain function: MoCA, MMSE tOCN: RIA	Higher tOCN associated with lower MoCA scores	Negatively
			No association between tOCN and MMSE scores	No correlation
^31^	n: 800 (90/710) Age: 76 Health Status: Community dwelling	Brain function: MMSE uOCN: measured by BML Inc.	Higher uOCN associated with impaired orientation, calculation and language	Negatively
^42^	n: 38 (8/30) Age: 57 Health Status: PHPT	Brain function: STAI‐S; BDI tOCN: RIA & ELISA	Lower tOCN associated with higher BDI and STAI‐S scores	Positively
^39^	n: 790 (87/703) Age: 76 Health Status: Community dwelling	Brain function: GDS uOCN: measured by BML Inc.	Higher uOCN associated with higher risk of depression	Negatively
^40^	n: 13 women Age: 50 Health Status: Depression	Brain function: MADRS uOCN: CAEIA	Lower uOCN associated with reduced depressive symptoms	Negatively
^43^	n: 158 (77/81) Age: Full‐term born Health Status: Vaginal births infants	Brain function: WPPSI‐III, ASQ‐III, MABC‐2, SDQ tOCN: the iSYS technique	Higher serum OCN at 4 months of age associated with higher intelligence quotient and better motor control at 4 years	Positively
			Higher cord OCN associated with poorer processing speed and fine motor control at 4 years of age	Negatively
^37^	n: 208 women Age: 49 Health Status: HIV infection and demographically similar HIV	Brain function: NP uOCN: ELISA	Higher uOCN associated with higher executive function in the total sample and in WLWH.	Positively
			Higher uOCN associated with higher motor skills in WLWH	Positively
			Higher uOCN associated with poorer attention/working memory in the total sample	Negatively
^44^	n: 120 (28/92) Age: 73 Health Status: POD and non‐POD	Brain function: CAM, DRS tOCN & uOCN: ELISA	Preoperative higher uOCN in CSF associated with a higher incidence of delirium and with greater severity of delirium	Negatively

*Note*: Association of osteocalcin with brain function outcomes – positively, higher osteocalcin associated with beneficial brain function; negatively, higher osteocalcin associated with detrimental brain function; no correlation, no correlation between osteocalcin and brain function.

Abbreviations: AD, Alzheimer's disease; ASQ‐III, the Ages & Stages Questionnaire, Third Edition; BDI, the beck depression inventory; CAEIA, commercially available enzyme immunoassay; CAM, the confusion assessment method; CANTB, the Cambridge Neurological Test Automated Battery; CMIA, commercial multiplex immunoassays; cOCN, carboxylated osteocalcin; DRS, the delirium rating scale‐98; ECLIA, electrochemiluminescence immunoassay; ELISA, enzyme‐linked immunosorbent assay; GDS, Japanese version of the 15‐item Geriatric Depression Scale; IGT, Iowa Gambling Task; MABC‐2, the Movement Assessment Battery for Children, Second Edition; MADRS, the Montgomery Asberg Depression Rating Scale; MMSE, mini mental state examination; MoCA, Montreal Cognitive Assessment; NP, neuropsychological test battery; OCN, osteocalcin; PHPT, primary hyperparathyroidism; PM, post‐menopausal; POD, postoperative delirium group; PSS, perceived stress scale; RBANS, the repeatable battery for the assessment of neuropsychological status; RIA, radioimmunoassay; SDQ, the Strengths and Difficulties Questionnaire; STAI‐S, State–Trait Anxiety Inventory‐State; T2DM, type 2 diabetes mellitus; tOCN, total osteocalcin; uOCN, undercarboxylated osteocalcin; WPPSI‐III, Wechsler Preschool and Primary Scale of Intelligence, Third Edition.

Eight studies examining the relationship between changes in osteocalcin blood concentrations and cognitive function were identified.^31^, ^32^, ^33^, ^34^, ^35^, ^36^, ^37^, ^38^ Six studies showed a single relationship, three showed a positive association, one showed a negative association, and two showed no association between high serum osteocalcin concentrations and cognitive function (Table 1).^31^, ^32^, ^33^, ^34^, ^36^, ^38^ A study of 800 elderly people in a community in Tokyo, Japan, reported that higher serum undercarboxylated osteocalcin concentrations were associated with impaired orientation, calculation, and language, that is, a negative correlation with brain function.^31^ The remaining two studies show more than one relationship. Pu et al. reported that high total osteocalcin was associated with lower montreal cognitive assessment scores but not mini mental state examination scores on cognitive tests in early‐stage men with Alzheimer's disease.^35^ In addition, Ross et al. reported differences in the association between cognitive scores and high undercarboxylated osteocalcin in human immunodeficiency virus‐infected patients and controls.^37^

Four studies reported an association between serum osteocalcin concentrations and depressive symptoms in patients.^39^, ^40^, ^41^, ^42^ One of the studies on primary hyperparathyroidism showed a positive correlation, reporting that low levels of osteocalcin were associated with poorer mental performance.^42^ Three other studies reported negative associations, two of which showed that elevated serum undercarboxylated osteocalcin levels were associated with an increased risk of depression.^39^, ^40^, ^41^

One study correlated serum osteocalcin with future neurodevelopment.^43^ Berggren et al. investigated the relationship between total serum osteocalcin levels and neurodevelopment at age 4 in 158 healthy full‐term vaginal infants.^43^ That is, increased serum osteocalcin levels at 4 months were associated with increased intelligence quotient and motor control at 4 years of age. In contrast, higher umbilical osteocalcin was associated with poorer processing speed and fine motor control at 4 years of age. Moreover, a recent study showed that high preoperative cerebrospinal fluid uncarboxylated osteocalcin concentrations were associated with the incidence and severity of postoperative delirium.^44^

There are many possible explanations for the inconsistent findings of previous studies of human osteocalcin and brain function. First, the methods used for measuring osteocalcin differ to some extent. Therefore, the use of standardized and reliable assays to report circulating concentrations of specific forms of osteocalcin is recommended in human studies.^45^, ^46^, ^47^ Different forms of osteocalcin are involved in different physiological processes on the body.^5^, ^14^ Therefore, it is not clear whether the ratio of osteocalcin is better than that of osteocalcin alone in reflecting the circulating concentration of patients.^48^ In previous clinical studies, the osteocalcin ratio has been shown to be a good predictor of bone loss and fracture risk.^49^, ^50^ Second, in some of the included studies, there were no reports that participants in the study might have used vitamin K, vitamin D, corticosteroids, exercise, or other factors known to affect circulating undercarboxylated osteocalcin levels.^2^, ^51^, ^52^, ^53^ Finally, the included studies recruited participants from different populations, including healthy individuals, patients of different age groups, individuals with diabetes, individuals with obesity, and individuals with other medical conditions. These risk factors have been reported to affect the serum osteocalcin concentration and may lead to inconsistent results.^48^, ^54^, ^55^ Overall, animal studies seem to indicate a protective effect of osteocalcin on central nervous system disease, which seems to contradict findings from some human biomarker studies. This may also be due to differences in osteocalcin between animals and humans.^8^ The nervous system can affect bone metabolism, and the positive association between high circulating osteocalcin and central nervous system disease in humans may be due to reverse causality.^56^, ^57^ Mendelian randomization studies may address the causal role of osteocalcin in central nervous system disease.^58^, ^59^

## 5 RECOMMENDATIONS FOR FUTURE RESEARCH

### 5.1 Bone and nervous system comorbidities

Osteocalcin is one of the mediators of bone and central nervous system communication. At the same time, by reviewing the literature, we found that some bone‐brain comorbidities are related to the mechanisms by which osteocalcin regulates brain function. Therefore, we will introduce the research on osteoporosis, multiple sclerosis, and cerebral palsy, and the potential association with osteocalcin to provide more breakthrough ideas for future research.

#### 5.1.1 Osteoporosis

Osteoporosis is a chronic systemic bone disease characterized by a decrease in bone mineral density and deterioration of bone microstructure and is accompanied by various neurological deficits such as cognitive impairment, anxiety, and depression.^60^, ^61^, ^62^ As osteocalcin is an important component of bone loss and bone formation, exploring the role of osteocalcin in osteoporosis, a systemic disease, will greatly promote our understanding of the physiology and pathology of the body.

Three observational studies are worth mentioning.^63^, ^64^, ^65^ First, patients with cognitive decline have an increased risk of bone loss and fracture.^63^ In contrast, another prospective study showed an improvement in memory in patients with cognitive decline after fracture rehabilitation.^65^ Although the association between osteocalcin and cognitive function was not mentioned in the above clinical studies, osteocalcin levels were significantly increased in patients with osteoporosis and Alzheimer's disease.^64^ These findings suggest that osteoporosis and Alzheimer's disease may have a common pathogenesis and that osteocalcin is involved in this pathogenesis. Of note, however, due to the presence of impaired bone formation per se in patients with osteoporosis, the use of other markers of bone turnover, in addition to the determination of different forms of osteocalcin, was suggested during the study.

#### 5.1.2 Multiple sclerosis

Multiple sclerosis is a chronic demyelinating disease characterized by intermittent episodes of focal inflammation and neurological dysfunction.^66^ Patients with multiple sclerosis are often complicated by bone loss, cognitive impairment, and depression.^67^ When these diseases occur together, they strongly affect daily activities and quality of life.

There are many potential links between osteocalcin and multiple sclerosis changes in the nervous system. First, brain‐derived neurotrophic factors play a neuroprotective role in multiple sclerosis.^68^ Radiographically, white matter damage in multiple sclerosis patients is associated with cognitive impairment.^69^, ^70^ Exercise training is a promising treatment for multiple sclerosis‐related cognitive impairment.^71^ Osteocalcin not only regulates brain function but also increases muscle mass and promotes exercise recovery.^7^, ^72^ Since only osteocalcin responds to bone formation in patients and there is no direct evidence linking changes in the nervous system to multiple sclerosis, future research could therefore begin by exploring the effects of osteocalcin on the nervous system in multiple sclerosis animal models.

#### 5.1.3 Cerebral palsy

Cerebral palsy is an umbrella term that encompasses a group of disorders characterized by impaired walking and is attributed to nonprogressive dysfunction of fetal and infant brain development.^73^ Dyskinesia in children with cerebral palsy is usually associated with neurological dysfunction and secondary musculoskeletal problems, and musculoskeletal interactions affect motor ability in children with cerebral palsy.^74^ People with cerebral palsy also experience bone loss due to impaired weight bearing, and the risk of fractures is even greater.^75^ Due to developmental delay and damage to various biological systems, people with cerebral palsy develop neurological disorders, including cognitive impairment, depression and anxiety, and pain, in childhood or early adulthood.^76^, ^77^, ^78^

Many of the mechanisms by which osteocalcin regulates neural function are also involved in cerebral palsy. First, in the transcriptional biomarkers of cerebral palsy, dysregulation of nutrient signaling pathways, such as brain‐derived neurotrophic factor pathways, is responsible for the decrease in neuronal protection.^79^ In terms of the molecular etiology of cerebral palsy, most patients have myelin dysfunction and abnormal lipid metabolism.^80^ In addition to regulating brain function, osteocalcin can also improve lipid metabolism.^81^ Second, enhancing the plasticity of children with cerebral palsy is beneficial for their rehabilitation.^82^ In a mouse model of cerebral palsy, modulation of the autophagy pathway prevents neuroinflammation and neuronal death.^83^ Different investigators have demonstrated that osteocalcin regulates the autophagy pathway.^23^, ^84^ Therefore, osteocalcin may have a promising future in further elucidating the etiology of abnormalities in different systems involved in cerebral palsy, such as the nervous and skeletal muscle systems.

### 5.2 Other future directions

#### 5.2.1 Myelin and OCN/GPR37

Myelin alterations and the OCN/GPR37 signaling pathway may be two good targets for further elucidating the osteocalcin‐related pathological progression of different neurological diseases (Figure 4A). First, Alzheimer's disease model mice exhibited increased myelin thickness similar to that observed in *OCN* ^*−/−*^ mice.^7^, ^85^ Recent studies in mice have shown that enhanced myelin turnover reverses cognitive impairment in Alzheimer's disease.^86^ Thus, studying aberrant changes in *OCN* ^*−/−*^ mice may contribute to elucidating the pathological progression of comorbidities of bone and the central nervous system, or to further understanding other mechanisms by which osteocalcin regulates cognition. Second, GPR37 has different functions in different neurological diseases.^87^, ^88^ Overexpression of the GPR37 receptor in Parkinson's disease stimulates autophagy in neurons.^87^ GPR37 is also overexpressed in neuropsychiatric diseases.^88^ Since osteocalcin regulates autophagy and anxiety‐like behavior, exploring the OCN/GPR37 signaling pathway is important for understanding the pathophysiological processes of neurological disorders (Figure 4B).

#### 5.2.2 A potential neuropeptide

We investigated whether osteocalcin plays other roles in the nervous system in addition to being an osteogenic hormone, such as by acting as a neuropeptide (Figure 4C). Researchers found reduced sensory responses in *OCN* ^*−/−*^ mice, suggesting that osteocalcin may act as a neuropeptide.^89^, ^90^ Moreover, immunoreactive neurons were found in both peripheral and central nerves.^91^, ^92^, ^93^, ^94^ Interestingly, a recent study on anxiety in OCN‐Cre mice showed that OCN‐Cre^+^ cells in the outer layer of the dorsal dentate gyrus of the hippocampus are selectively activated to exert anxiolytic effects upon exposure to an external anxiety‐inducing stimulus.^95^ OCN‐Cre transgenic mice were generated by gene knockout technology using the human osteocalcin gene promoter (see “Materials and Methods” in the literature).^96^ Although the above study suggested that osteocalcin may be a potential neuropeptide, it should be ruled out that osteocalcin is a marker of only neurons. Both studies showed that osteocalcin‐knockout mice had hypoesthesia, but the hypoesthesia in one of the studies may be due to myelin alterations.^7^, ^89^ Therefore, whether osteocalcin functions as a neuropeptide needs to be explored carefully.

## 6 CONCLUSION

After the synthesis of osteocalcin, due to the different carboxylation conditions, mainly uncarboxylated osteocalcin, undercarboxylated osteocalcin and decarboxylated osteocalcin are present in the circulation. The main functions of osteocalcin in the central nervous system include its involvement in neural structure and neuroprotection and its regulation of cognition and anxiety. In diseases of the central nervous system, good positive results have been obtained in animal studies, i.e., osteocalcin improves cognitive function. In some human studies, high osteocalcin has been associated with improved brain function (i.e., cognitive function, depression, neurodevelopment). However, there have been some different findings in human studies, such as some studies linking high osteocalcin with deterioration of brain function. One explanation for this is reverse causality, in which high levels of osteocalcin in patients with brain dysfunction may be a response to the disease rather than a cause. At the same time, osteocalcin, a member of the bone‐brain axis, is speculated to play a promising role in exploring the pathogenesis of bone‐nervous system comorbidities. In addition, the mechanisms related to the regulation of brain function by osteocalcin, multiple sclerosis, and cerebral palsy were clarified. Future research directions for osteocalcin in the regulation of brain function, namely, myelin changes and the OCN/GPR37 signaling pathway, are suggested, and osteocalcin may be a potential neuropeptide.

## AUTHOR CONTRIBUTIONS

X.S.Q. wrote the first draft of the manuscript. X.S.Q., X.H., Y.P., X.H.H., Q.Y.Y., K.J., and H.L. contributed to writing, correction, and addition of fundamental insights to the manuscript. H.L. and K.J. conceived the presented idea, organized the structure of the review, and took the lead in writing the manuscript. All authors read and approved the final version of the manuscript.

## FUNDING INFORMATION

This work was supported by the Young Outstanding Scientific and Technological Talent of Guizhou Province (grant No. Qiankehepingtairencai[2021]5620), Key Basic Research Program of Guizhou Province (grant No. Qiankehejichu‐ZK[2022]zhongdian051), and Talent Program for Future Famous Clinical Doctors of Zunyi Medical University (rc220211205).

## CONFLICT OF INTEREST STATEMENT

All authors state that they have no competing interests.

## Data Availability

Not applicable.
